# Commentary: Risk factors and early markers for echovirus type 11 associated haemorrhage-hepatitis syndrome in neonates, a retrospective cohort study

**DOI:** 10.3389/fped.2024.1338097

**Published:** 2024-03-25

**Authors:** Haider Al-Hello, Soile Blomqvist, Carita Savolainen-Kopra

**Affiliations:** ^1^ Expert Microbiology Unit, Department of Health Security, National Institute for Health and Welfare, Helsinki, Finland; ^2^ College of Health and Medical Technologies, National University of Science and Technology, Thi-Qar, Iraq

**Keywords:** mutations, RNA-dependant RNA polymerase, echovirus 11, enterovirus, fidelity

A Commentary on Risk factors and early markers for echovirus type 11 associated haemorrhage-hepatitis syndrome in neonates, a retrospective cohort study By Wang P, Xu Y, Liu M, Li H, Wang H, Liu Y, Wang B, Xia S, Su H, Wei M, Tao L, Chen X, Lu B, Gu X, Lyu H, Zhou W, Zhang H, Gong S (2023). Front. Pediatr. 11:1063558. doi: 10.3389/fped.2023.1063558

Dear Editors

We have read with great interest the publication by Wang et al. (1), which reports on haemorrage-hepatitis syndrome in neonates caused by Echovirus 11 (E-11). We appreciate the article's suggestions on the diagnosis and treatment of haemorrage-hepatitis syndrome but wonder if the data and conclusions regarding the similarity of the E-11 strain isolated in 2019 GWCMC01/GZ/CHN/2019 and E-11 D207 (GenBank accession EF634316), isolated in 2002 and not in 2007, as stated in the publication above, should need further study. The reasons are as follows:

Firstly, the RNA-dependent RNA polymerase of the picornavirus family has low replication fidelity, and the absence of proofreading mechanism leads to genetic mutations. The mutation rate for RNA viruses is 10^−3^ to 10^−5^ per 10 kb of the RNA genome, which is equivalent to 0.1–10 mutations (for example, the echovirus 11 may have a mutation for each transcript). The mutation rate of polioviruses, based on several studies, is approximately 3 × 10^−2^ mutations/synonymous site/year in the gene encoding viral protein 1 (VP1) (2). As a result of this high error rate, each time the genome is replicated, new variants arise that produce heterogeneous virus progeny referred to as “quasispecies” (3–5). High mutation frequencies allow these viruses to rapidly adapt to changing environments. According to information obtained from GenBank, the two strains carry only four nucleotide changes throughout the genome. These mutations are located in the 5′ untranslated region (5′UTR) of the genome and thus the entire capsid coding region as well as non-structural proteins coding region are identical. The high similarity between the two virus strains is challenging to understand, considering that the time between the first and the second isolations was 18 years, and the geographical area was completely different.

Secondly, according to our article published in 2007, the E-11 D207 (EF634316) is pancreatropic. This means that E-11 D207 tends to replicate in the pancreatic islets of Langerhans, which was documented in association with diabetes in a Slovakian child. To gain new phenotypic features, the virus is supposed to acquire a new genetic characteristic represented by nucleotide mutations and amino acid substitutions. In the case of the publication Al-Hello et al. (6), the pancreatropic E-11/D207 was found to be closely related to a specific subgroup B of E-11 strains known to cause uveitis. The strains are E-11/Kust/86, E-11/Kar/87 and E11/Kh3/97 published by Lukashev et al. (7, 8). The first two strains were causative agents of uveitis outbreaks in Siberia in 1986 and 1987, while the third virus was an occasional isolate in the Russian Far East in 1997. The genetic similarity between uveitis strains and E-11 D207 (EF634316) ranges from 90% to 91%. Although they were isolated in Russia and Siberia over a similar or shorter period of time as compared to the period of time between E-11 D207 (EF634316) and GWCMC01/GZ/CHN/2019, the genetic similarity is much less.

Thirdly, in enterovirus species B (EV-B) recombination and mutations caused by the error-prone polymerase lacking proofreading machinery have been recognized as the main mechanisms of evolution (2, 4). EV-B, to which echovirus 11 belongs, is the most abundant species of enterovirus. Recombination events have been detected mostly in species B (9). Comparison of phylogenetic trees from different genomic regions has revealed recombination events in several types, such as E-7, E-30, E-11, E-9, and CV-B (10–14). In addition, interspecies recombination has also been observed, such as what occurs in the 5′UTR in EV-A and EV-B (15, 16). This indicates that the process of recombination among enteroviruses is abundant in nature, and for this reason, the high similarity between the two strains with long time period between them is difficult, if not impossible, to understand.

For the above mentioned reasons, we would like to draw your attention to the fact that this high similarity between the two strains is highly unlikely. Also, we would like to inform you that GWCMC01/GZ/CHN/2019 virus strain has been used in several studies (17–19), which raises a concern about the results of those and potential new studies, if genomic data is used to draw conclusions. It is critical to note that we approached this issue from a virological perspective, and we do not take a stance on the other clinical aspects and patient care described in the paper. However, for E-11 inducing diabetes or hemorrhage-hepatitis, it is necessary to improve prevention, facilitate treatment and avoid further spread.

Sincerely,

Haider Al-Hello, Soile Blomqvist and Carita Savolainen-Kopra
