# Corrigendum: Commentary: Risk factors and early markers for echovirus type 11 associated haemorrhage-hepatitis syndrome in neonates, a retrospective cohort study

**DOI:** 10.3389/fped.2025.1642238

**Published:** 2025-06-20

**Authors:** Haider Al-Hello, Soile Blomqvist, Carita Savolainen-Kopra

**Affiliations:** ^1^ Expert Microbiology Unit, Department of Health Security, National Institute for Health and Welfare, Helsinki, Finland; ^2^ College of Health and Medical Technologies, National University of Science and Technology, Thi-Qar, Iraq

**Keywords:** mutations, RNA-dependant RNA polymerase, echovirus 11, enterovirus, fidelity

A Corrigendum on Commentary: Risk factors and early markers for echovirus type 11 associated haemorrhage-hepatitis syndrome in neonates, a retrospective cohort study By Al-Hello H, Blomqvist S, Savolainen-Kopra C (2024). Front Pediatr. 12:1338097. doi. 10.3389/fped.2024.1338097

**Additional affiliation(s)**

In the published article, there was an error regarding the affiliation(s) for [Haider Al-Hello]. As well as having affiliation(s) [1], they should also have [College of Health and Medical Technologies, National University of Science and Technology, Thi-Qar, Iraq].

We apologize for this error and we confirm that this does not change the scientific conclusions of the article in any way. The original article has been updated.

